# *Malva pseudolavatera* Leaf Extract Promotes ROS Induction Leading to Apoptosis in Acute Myeloid Leukemia Cells In Vitro

**DOI:** 10.3390/cancers12020435

**Published:** 2020-02-13

**Authors:** Marianne El Khoury, Tony Haykal, Mohammad H. Hodroj, Sonia Abou Najem, Rita Sarkis, Robin I. Taleb, Sandra Rizk

**Affiliations:** 1 Department of Natural Sciences, Lebanese American University, Byblos 1401, Lebanon; marianne.elkhoury01@lau.edu (M.E.K.); tony.haykal@lau.edu (T.H.); mohammadhassan.hodroj@lau.edu (M.H.H.); sonia.abounajem@lau.edu.lb (S.A.N.); robin.taleb@lau.edu.lb (R.I.T.); 2 Laboratory of Regenerative Hematopoiesis, Swiss Institute for Experimental Cancer Research (ISREC) & Institute of Bioengineering (IBI), School of Life Sciences, Ecole Polytechnique Fédérale de Lausanne (EPFL), 1015 Lausanne, Switzerland; Rita.sarkis@epfl.ch

**Keywords:** *Malva pseudolavatera* Webb & Berthel., apoptosis, acute myeloid leukemia, reactive oxygen species

## Abstract

*Malva pseudolavatera* Webb & Berthel. is a plant from the Malvaceae family that has long been included in the human diet due to its various curative effects. Many plant leaf extracts from the various species of *Malva* genus have been reported to possess anti-cancer properties, however, studies on *M. pseudolavatera* Webb & Berthel. leaves have documented anti-inflammatory and anti-oxidant effects with no emphasis on their possible anti-cancer potential. The present study explores the anti-cancer properties of *Malva pseudolavatera* Webb & Berthel. leaf extract on acute myeloid leukemia (AML) cell lines in vitro and deciphers the underlying molecular mechanism. Treatment of AML cell lines with *M. pseudolavatera* methanolic leaf extract showed a dose- and time-dependent inhibition of proliferation and a dose-dependent increase in apoptotic hallmarks such as an increase in phosphatidylserine on the outer membrane leaflet and membrane leakage in addition to DNA fragmentation. The pro-apoptotic effect was induced by reactive oxygen species (ROS) as well as an upregulation of cleaved poly(ADP-ribose) polymerase (PARP), increase in Bax/Bcl-2 ratio, andrelease of cytochrome-c from the mitochondria. Major compounds of the extract included methyl linolenate, phytol, γ-sitosterol, and stigmasterol as revealed by gas chromatography coupled with mass spectrometry, and amino acids, amino acid derivatives, tiliroside, 13-hydroxyperoxyoctadecadienoic, and quercitrin as detected by liquid chromatography coupled to mass spectrometry.

## 1. Introduction

*Malva pseudolavatera* Webb & Berthel. is an annual or biennial subshrub that grows in fields and roadsides in coastal areas and low-altitude mountain regions [1]. Commonly known as “tree mallow” in North America and “khubbaza” in the Middle East, it is a plant of the Malvaceae family [2]. Previously named *Lavatera cretica* (Malvaceae family), the species was transferred to the *Malva* genus and is currently called *M. pseudolavatera* or *Malva linnaei* or *Malva multiflora* [3]. *Malva pseudolavatera* Webb & Berthel. is the accepted name of the species as included on http://www.theplantlist.org [4].

Apart from being used as food in some regions such as in Turkey, Spain, and Pakistan, leaves of the *Malva* genus plants have been traditionally used in folk medicine all around the world to treat a multitude of diseases, most commonly diarrhea, arthritis, inflammation, cough, and respiratory infections [5]. Among the 50 species of Malva, *M. pseudolavatera* Webb & Berthel. is known for its versatile uses [6]. In Spain, it is considered as a remedy for influenza, upper respiratory tract infections, and cough [7], whereas in Portugal, it is used for its laxative, analgesic, and antiseptic effects [8]. In fact, studies showed that *M. pseudolavatera* Webb & Berthel. aqueous leaf extracts were able to scavenge free radicals and inhibit lipoxygenase activity in vitro, indicating its potent antioxidant and anti-inflammatory activities [9].

Malva species leaves have a lot of similarities in the overall morphology and anatomy, and only differ in some characteristics such as the number of lobes, size of the blade, or margin dentation. Leaves of many Malva species such as *M. sylvestris* and *M. parviflora* share a common basic chemical composition as they all contain anthocyanins, flavonoids, essential oils, and tocopherols [10,11]. In fact, many studies have shown the inhibitory effects of leaf extracts from Malva species on cancer cell lines. The methanolic extract from *M. sylvestris* leaves exhibited a dose-dependent cytotoxicity on melanoma and lymphoma cell lines in vitro [12]. Moreover, the ethanolic extract from *M. parviflora* leaves showed mild cytotoxicity against the MCF-7 human breast adenocarcinoma cell line [13]. Other studies have reported that the hexane and methanol extracts from *M. parviflora* leaves inhibited the proliferation of HeLa cervical carcinoma cells, but the aqueous extract did not cause any inhibition of cell proliferation [14]. However, no studies have examined the anti-cancer potential of extracts from *M. pseudolavatera* Webb & Berthel. leaves.

Acute myeloid leukemia (AML) is a type of cancer that starts in the bone marrow and quickly moves undifferentiated myeloblasts into the blood [15]. It is an aggressive malignancy with incidence levels still on the rise in several countries including Canada, UK, and Australia [16]. Chemotherapy is the main treatment for AML [17] and plants have long been used as important sources for novel chemotherapeutic drug characterization [18,19].

In the present study, we investigated the potential anti-cancer properties of *M. pseudolavatera* Webb & Berthel. methanolic leaf extract (MMLE) on AML cell lines in vitro and deciphered the underlying molecular mechanism.

## 2. Materials and Methods

### 2.1. Cell Culture

Acute myeloid leukemia cell lines, namely Mono-Mac-1, U937, and KG-1 (American Type Culture Collection), were cultured in Roswell Park Memorial Institute medium (RPMI, Sigma-Aldrich, St. Louis, MO, USA)supplemented with 10% fetal bovine serum (FBS) (Gibco^TM^, Dublin, Ireland) and 100 U/mL penicillin and 100 µg/mL streptomycin (Lonza, Basel, Switzerland) in a humidified incubator at 5% CO_2_ at 37 °C. Trypan blue exclusion method was used to count the cells before experimentation.

### 2.2. Isolation and Culture of Mesenchymal Stem Cells (MSCs) from Rat Bone Marrow

A single, 12-week-old rat was provided by the animal facility at the Lebanese American University. The animal was maintained under optimal laboratory conditions and received food and water ad libidum. All experiments were approved by the university’s Animal Care and Use Committee (ACUC) and complied with the Guide for the Care and Use of Laboratory Animals (Committee for the Update of the Guide for the Care and Use of Laboratory Animals, 2010) [20,21]. MSCs were isolated from rat bone marrow according to a modified procedure. Briefly, the rat was sacrificed by CO_2_ asphyxiation and both hind legs were aseptically removed. Femoral and tibial bones were then isolated and washed with 70% ethanol and placed in sterile phosphate buffered saline (PBS, Lonza) supplemented with 100 U/mL penicillin and 100 µg/mL streptomycin (Lonza). After removing the bone epiphyses with sterilized scissors, the bone marrows were flushed out using a needle filled with Dulbecco’s modified Eagle medium (DMEM, Sigma-Aldrich) supplemented with 10% fetal bovine serum (Gibco^TM^) and 100 U/mL penicillin and 100 µg/mL streptomycin (Lonza). The cells collected were then incubated in vented flasks at 37 °C with 5% CO_2_. After 5 days of daily medium change, MSCs were identified by their spindle-shaped morphology as observed using the ZOE fluorescent cell imager (Bio-Rad, Irvine, CA, USA) [22,23,24].

### 2.3. Isolation and Culture of Normal Mononuclear Cells (MNCs) from Human Bone Marrow (BM)

Normal mononuclear cells were offered by Dr. Marwan El-Sabban’s Lab at the American University of Beirut (AUB) as a kind gift. The normal MNCs were obtained originally from bone marrow aspirate leftovers of healthy patients attending AUB Medical center (AUB-MC). BM aspirates were centrifuged on Ficoll/Hypaque (GE Healthcare Life Sciences, Uppsala, Sweden), a density gradient step to separate MNCs from red blood cells and neutrophils. Then the buffy coat was aspirated and seeded in petri dishes using Dulbecco’s modified Eagle’s medium (DMEM)-low glucose (Sigma-Aldrich, Saint Louis, MO, USA) supplemented with 10% FBS (Gibco, Dublin, Ireland) and 100 U/mL penicillin and 100 µg/mL streptomycin (Lonza, Basel, Switzerland) in a humidified incubator at 37 °C and 5% CO_2_. One week later, the cells in suspension were collected as a purified MNC population and cultured in the same conditions as detailed by Zibara et al. [25].

### 2.4. Plant Material

*Malva pseudolavatera* Webb & Berthel. leaves were collected from Batroun, Lebanon (34.2498° N, 35.6643° E. 20 m above sea level), during January 2018, and identified according to the indications and characteristics described by Edgecombe [2], and then identified by Dr. Nisrine Machaka-Houri, plant researcher and expert on Lebanese flora [26]. A voucher specimen was deposited in the Beirut Arab University Herbarium (ID-RCED2019-361).

### 2.5. Preparation of Crude Leaf Extract (MMLE)

Leaves were washed with distilled water, stored between paper towel sheets at 4 °C for 2 weeks to dry out, then ground and left to shake in absolute methanol at 200 rpm for 1 week. The extract was later filtered through a cheesecloth and centrifuged at 15,000 rpm to discard the pellet. Methanol was evaporated using a rotary evaporator. The methanolic crude extract was weighed, then dissolved in dimethyl sulfoxide (DMSO) and diluted with RPMI to a final concentration of 9 mg/mL. When applied on the cell lines, the DMSO level maximally reached 0.8% at 360 μg/mL for KG-1 and Monomac-1 and 1% at 450 μg/mL for U937.

### 2.6. Cytotoxicity Assay

AML cells and MSCs were seeded in 96-well plates at a density of 0.5 × 10^5^ cells/well and incubated overnight before treatment of triplicates of wells with increasing concentrations of *M. pseudolavatera* Webb & Berthel. methanolic leaf extract (MMLE). After 24 or 48 h of incubation, WST-1 cell proliferation reagent (Roche, Mannheim, Germany) was used to estimate cell viability according to the manufacturer’s guidelines. Absorbance of each well was detected at 450 nm using a Multiskan^TM^ FC microplate photometer to quantify metabolically-active cells before calculating the percent proliferation relative to the control untreated cells.

### 2.7. Cell Cycle Analysis

Monomac-1 cells were seeded in 6-well plates at a density of 1 × 10^5^ cells/well and incubated overnight before treatment with increasing concentrations of MMLE for 24 h. Cells were then fixed overnight with ethanol, and the DNA was stained with propidium iodide (PI, Sigma-Aldrich) after the enzymatic removal of RNA using RNase (Roche). DNA content was measured using an Accuri C6 flow cytometer to determine the distribution of cells in each cell cycle phase: pre-G0/G1 phase cells had <2n, G0/G1 phase cells had 2n, S phase cells had between 2n and 4n, and G2/M phase cells had 4n.

### 2.8. Apoptosis Detection Using Fluorescent Annexin V Staining

Monomac-1 and KG-1 cells were seeded in 24-well plates at a density of 1 × 10^5^ cells/well and incubated overnight before treatment with increasing concentrations of the MMLE for 24 h. Cells were then stained with annexin V-FITC (Abcam, Cambridge, UK)and visualized with the ZOE fluorescent cell imager under bright-field conditions then the filter was set for FITC before merging the images.

### 2.9. Apoptosis Quantification by Dual Annexin V/PI Staining

Monomac-1 cells were seeded in 6-well plates at a density of 2 × 10^5^ cells/well and incubated overnight before incubation with increasing concentrations of the MMLE for 24 h. Cells were then stained with annexin V-FITC and PI (Abcam) according to the manufacturer’s instructions and analyzed by the Accuri C6 flow cytometer. Annexin V binds to phosphatidylserine molecules translocated to the outer layer of the cell membrane upon apoptosis induction. PI reaches the cellular DNA in cells that have lost the cellular membrane integrity, so it stains late apoptotic and necrotic cells but not viable and early apoptotic cells. This allowed for the discrimination between viable, early apoptotic, late apoptotic, and necrotic cells. 

### 2.10. Cell Death ELISA

Monomac-1 and KG-1 cells were seeded in 24-well plates at a density of 2 × 10^5^ cells/well and incubated overnight before treatment of duplicates of wells with increasing concentrations of MMLE. Treatment with the chemotherapeutic drug, etoposide (Abcam) at a concentration of 100 μM (58.85 μg/mL) was used as positive control. After 24 h, cells were collected and lysed in incubation buffer before quantification of fragmented cytosolic histone-associated-DNA content using the Cell Death ELISA kit according the manufacturer’s instructions (Roche). Extracted DNA was then incubated in wells coated with biotin-associated anti-histone antibodies, followed by incubation with anti-DNA antibodies linked to peroxidase enzyme, then washed with washing buffer before the addition of the peroxidase substrate. Absorbance at 405 nm was measured by spectrophotometry using a Multiskan^TM^ FC microplate photometer and the DNA fragmentation enrichment factor (absorbance of treated cells/absorbance of non-treated cells) was calculated as the ratio of absorbance in the treated samples to that of the untreated controls.

### 2.11. Western Blot

Monomac-1 cells were plated in 6-well plates at a density of 5 × 10^5^ cells/mL before treatment with two increasing concentrations of MMLE for 24 h. The concentrations used were the closest to the IC50. Total proteins were extracted using the Qproteome mammalian protein prep kit (Qiagen, Hilden, Germany) and quantified using the Lowry method. Proteins were then separated by SDS-PAGE (10%) and transferred to PVDF membranes that were blocked with 5% skimmed milk, then incubated with primary antibodies: anti-β-actin (Santa Cruz Biotechnology, Dallas, Tx, USA), anti-cytochrome-c and anti-cleaved poly(ADP-ribose) polymerase (PARP) (Abcam), anti-Bax and anti-Bcl2 (Elabscience, Houston, TX, USA). β-actin was used as a loading control. Membranes were then washed and incubated with a secondary antibody (Bio-Rad, Irvine, CA, USA) followed by exposure for image development using Clarity™ Western ECL substrate (Abcam) on a ChemiDoc machine (Bio-Rad). Quantification using the ImageJ program allowed us to calculate the relative expression of proteins, as compared to the loading control.

### 2.12. Reactive Oxygen Species Detection

Using the DCFDA cellular ROS detection assay kit (Abcam), levels of ROS were quantified in Monomac-1 and KG-1 cells treated with increasing concentrations of MMLE. Tert-butyl hydrogen peroxide (TBHP) is a potent ROS inducer and was used as a positive control. DCFDA (2′,7′-dichlorodihydrofluorescein diacetate) oxidative conversion to H_2_DCFDA upon ROS reduction was quantified by spectrofluorometry on the Varioskan™ LUX multimode microplate reader (Thermo Fisher Scientific, Bremen, Germany).

### 2.13. Gas ChromatographyMass Spectrometry Analysis of the Methanolic Extract of M. Pseudolavatera Webb & Berthel. Leaves

MMLE composition was analyzed using gas chromatography coupled with mass spectrometry (GC-MS). The carrier gas used was helium with splitless injection and a flow rate of 1.2 mL/min was applied. A temperature program consisted of 2 min at 70 °C, from 70 °C to 130 °C at 8 °C/min and hold for 5 min, from 130 °C to 180 °C at 2 °C/min and hold for 10 min, from 180 °C to 220 °C at 15 °C/min and hold for 2 min, and then from 220 °C to 280 °C at 15 °C/min and hold for 22 min. Preliminary identification of the various compounds was performed by comparing their mass spectra with the literature (NIST11 and Wiley9). Percentage composition was computed from GC peak areas.

### 2.14. Liquid ChromatographyMass Spectrometry Analysis of the Methanolic Extract of M. Pseudolavatera Webb & Berthel. Leaves

A 2.5 µg sample was injected into C18 Gravity-SB Nucleodur (300 Å, 1.8 µm, 2 × 100 mm, Macherey-Nagel, Düren, Germany) using a Dionex Ultimate 3000 analytical RSLC system (Dionex, Germering, Germany) coupled to a heated electrospray source HESI source (Thermo Fisher Scientific, Bremen, Germany). The separation was performed with flow rate of 300 µl/min by applying a gradient of solvent B from 3% to 50% within 35 min, followed by column washing and re-equilibration steps. Solvent A was composed of water with 0.1% formic acid, while solvent B consisted of acetonitrile with 0.1% formic acid. Eluting compounds were analyzed on a QExactive HF-HT-Orbitrap-FT-MS benchtop instrument (Thermo Fisher Scientific, Bremen, Germany). MS1 scan was performed with 60,000 resolution, AGC (automatic gain control) of 3e6 and maximum injection time of 200 ms. MS2 scan was performed in Top10 mode with 2 m/z isolation window, AGC of 5e5, 15 000 resolution, maximum injection time of 50 ms, and averaging 2 µscans. Higher-energy collisional dissociation (HCD) was used as the fragmentation method with normalized collision energy of 28%. For compound analysis, mzCloud and ChemSpider database for chemicals were used.

### 2.15. Statistical Analysis

All experiments were repeated three times (n=3). Statistical analyses were performed using GraphPad Prism 8. The data was reported as mean ± SEM and the *p*-values were calculated by *t*-tests or two-way ANOVA depending on the experiment. Significant differences were reported with * indicating a *p*-value of 0.01 < *p* < 0.05, ** indicating a *p*-value of 0.001 < *p* < 0.01, *** indicating a *p*-value of 0.0001 < *p* < 0.001, and **** indicating a *p*-value of *p* < 0.0001.

## 3. Results

### 3.1. M. pseudolavatera Leaf Extract Exhibits Selective Anti-Proliferative Effects on AML Cell Lines

**Figure 1:**
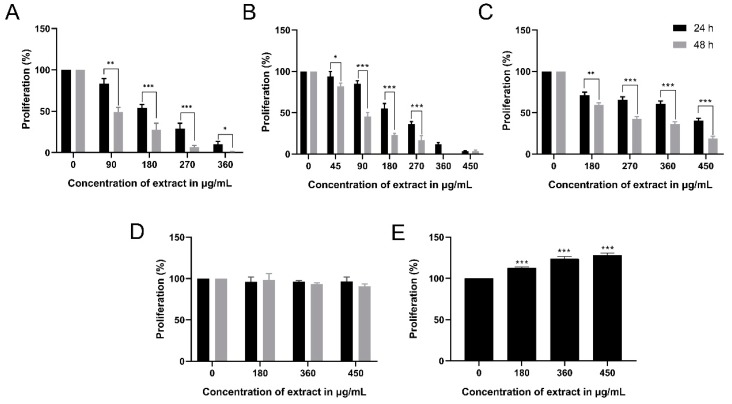
Proliferation of Monomac-1 (**A**), KG-1 (**B**), U937 (**C**), mesenchymal stem cells (MSCs) (**D**), and normal mononuclear cells MNCs (**E**), after 24 h and 48 h of treatment with methanolic leaf extract (MMLE). A significant dose- and time-dependent inhibition of proliferation of the three AML cell lines was noticed with increasing concentrations of MMLE. Significant differences were reported with * indicating a *p*-value: 0.01 < *p* < 0.05, ** indicating a *p*-value: 0.001 < *p* < 0.01 and *** indicating a *p*-value: 0.0001 < *p* < 0.001.

In order to detect the percent proliferation of AML cell lines, MSCs, and MNCs treated with MMLE, WST-1 cell proliferation reagent was used. A dose-dependent and time-dependent significant decrease in proliferation of the three AML cell lines, Monomac-1, KG-1, and U937 was observed with an IC50 of 200 μg/mL and 86.80 μg/mL for Monomac-1 (Figure 1A), 207.9 μg/mL and 89.47 μg/mL for KG-1 (Figure 1B), and 402 μg/mL and 229 μg/mL for U937 (Figure 1C) after 24 h and 48 h, respectively. The extract had no significant cytotoxic effect on MSCs and MNCs (Figure 1D,E). This indicates that MMLE exhibits selective anti-proliferative effects on all AML cancer cell lines used.

### 3.2. M. pseudolavatera Leaf Extract Induces Cellular Fragmentation in AML Cell Lines

**Figure 2:**
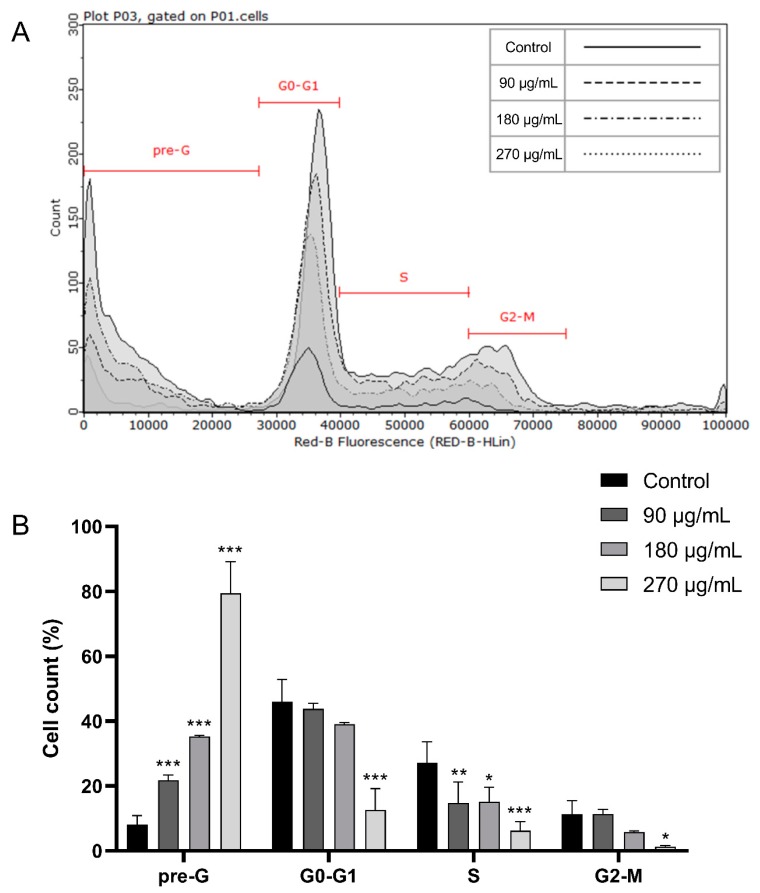
Cell cycle analysis of Monomac-1 treated with MMLE for 24 h (**A**). The percentage of the cells in different phases of cell cycle was determined by C Flow software (**B**) A significant increase in the pre-G and a decrease in G0/G1, S, and G2/M in a dose-dependent manner was obtained and indicated an increase in DNA fragmentation in Monomac-1 cells upon MMLE treatment.

In order to elucidate the mechanism by which MMLE exerted its cytotoxic effect, PI staining was performed. To check for any cell cycle arrest and analyze the cell cycle distribution of Monomac-1 cells treated with MMLE, DNA content was quantified by PI staining followed by cytometric analysis. A dose-dependent increase in cellular fragmentation was detected as the cells gradually shifted from the G0/G1, S, and G2/M stages to the pre-G0/G1 stage where cells are fragmented and contain DNA <2n. In fact, the proportion of Monomac-1 cells in the pre-G0/G1 stage increased significantly from 8.05% in the untreated cells to 74.9% in cells treated with 270 μg/mL (after IC50) (Figure 2). This shows that MMLE does not induce a cell cycle arrest, but rather activates a mechanism leading to cellular fragmentation.

### 3.3. M. pseudolavatera Leaf Extract Significantly Induces Apoptosis in AML Cell Lines

**Figure 3:**
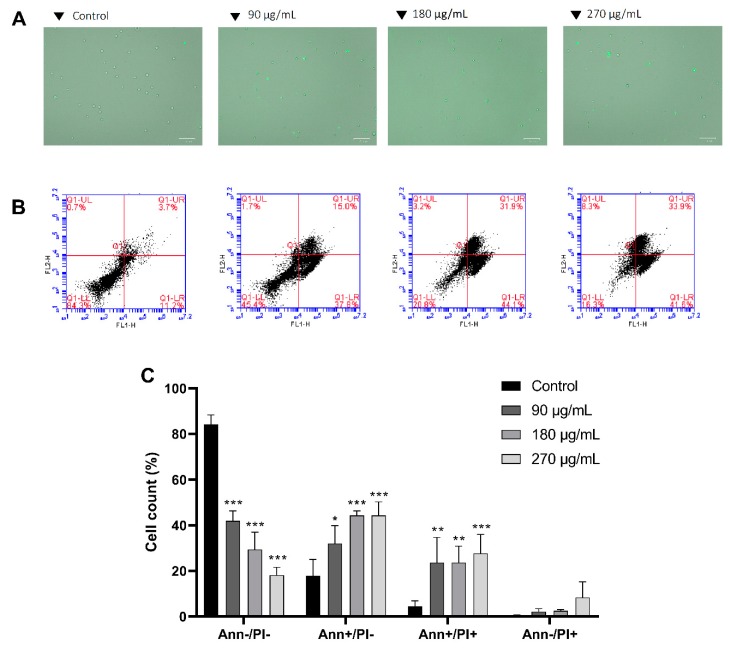
Annexin V (**A**) and annexin V/PI staining (**B**,**C**) of Monomac-1 cells treated for 24 h with increasing concentrations of MMLE. A significant increase in positively stained cells was observed upon 24 h treatment with increasing concentrations of MMLE (A). A decrease in annexin-negative/PI-negative stained cells and an increase in annexin-positive/PI-negative, annexin-positive/PI-positive, and annexin-negative/PI-positive stained cells were noted in Monomac-1 treated with 90, 180, and 270 μg/mL for 24 h.

To explore whether cell death is induced by apoptosis, annexin V staining was followed by fluorescence microscopy. Upon treatment with increasing concentrations of MMLE, a marked increase in annexin binding on Monomac-1 cells was observed, indicating a shift of phosphatidylserine from the inner leaflet to the outer leaflet of the cell membrane, a major apoptotic event (Figure 3A).

To quantitatively assess the induction of apoptosis, flow cytometry analysis was carried out after annexin V/PI staining and cells were distributed into four quadrants where the lower left quadrant represents normal healthy cells, negatively staining for both annexin V and PI. The lower right quadrant represents early apoptotic cells which stain positively only for annexin V. The upper right quadrant represents the late apoptotic cells, staining positively for both annexin V and PI. The upper left quadrant represents necrotic cells which stain positively for PI only. After 24 h of treatment with increasing concentrations of MMLE, a decrease in Monomac-1 healthy cells from 84.3% in the control group to 18.2% at 270 μg/mL was coupled to a significant increase in early apoptotic cells from 17.8% in the control to 44.9% when treated with 270 μg/mL MMLE. A significant increase in late apoptotic cells from 4.5% to 27.7% was also observed upon treatment (Figure 3B,C). This shows that apoptosis is the likely mechanism by which MMLE inhibits the proliferation of AML cell lines. 

**Figure 4:**
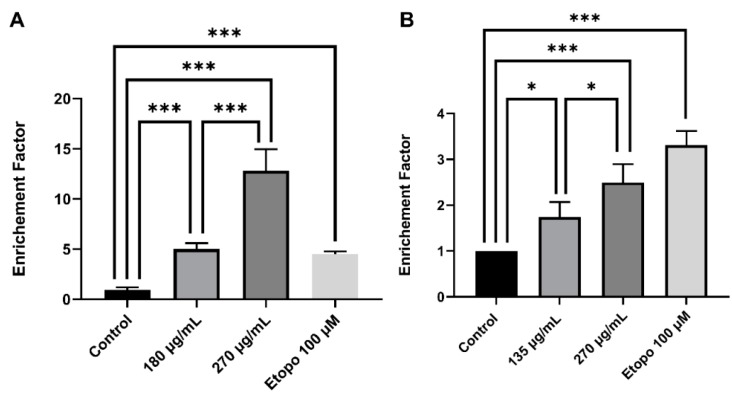
Cell Death ELISA on Monomac-1 (**A**) and KG-1 cells (**B**) treated with increasing concentrations of MMLE and a positive control treated with etoposide for 24 h. A significant dose-dependent increase in the enrichment factor was observed in both Monomac-1 and KG-1 cells when treated with concentrations before and after IC50. Significance relative to the control was reported with * indicating a *p*-value: 0.01 < *p* < 0.05 and *** indicating a *p*-value: 0.0001 < *p* < 0.001.

To validate apoptosis induction, DNA fragmentation was quantified by Cell Death ELISA kit. A 5.0-fold and 12.8-fold significant increase in DNA fragmentation was observed upon 24 h treatment of Monomac-1 with 180 μg/mL and 270 μg/mL, respectively (Figure 4A), which correspond to concentrations below and above the IC50. A similar pattern of DNA fragmentation was noted in KG-1 cells with a 1.7-fold and 2.5-fold increase after 24 h treatment with 135 μg/mL and 270 μg/mL respectively (Figure 4B). The increase of the dual stain fluorescence using annexin V/PI dual staining and DNA fragmentation confirms that MMLE induces apoptosis in Monomac-1 and KG-1 cell lines in a dose-dependent manner.

### 3.4. M. pseudolavatera Leaf Extract Causes Upregulation of Pro-Apoptotic Proteins

**Figure 5:**
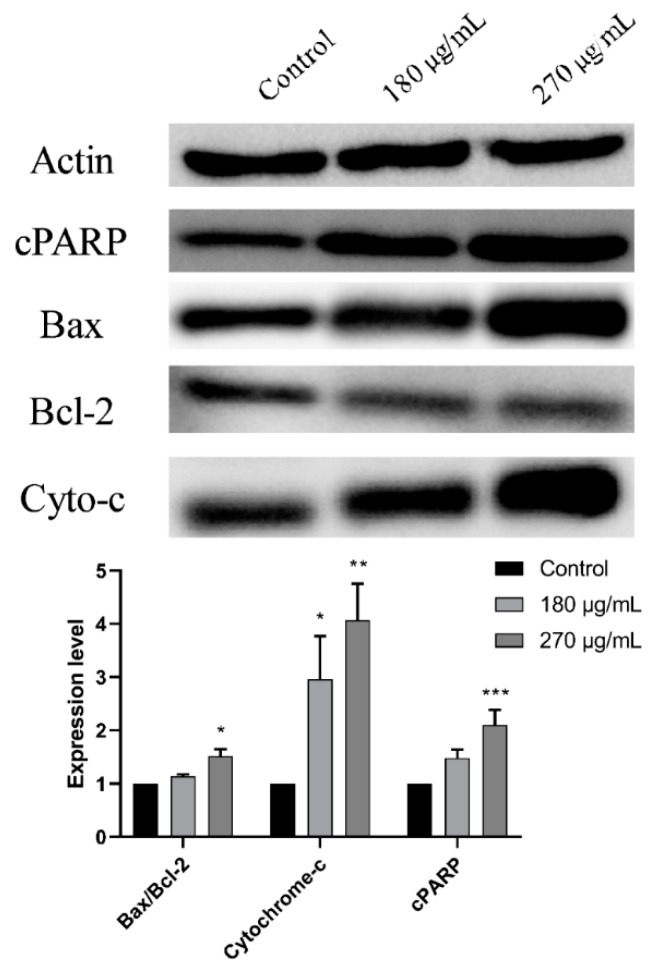
Western blot analysis and quantification of expression levels of apoptosis-regulating proteins in Monomac-1 cells treated with MMLE for 24 h. Significant upregulation of pro-apoptotic proteins such as cleaved poly(ADP-ribose) polymerase (PARP), Bax, and cytochrome-c and downregulation of anti-apoptotic proteins such as Bcl-2 were observed upon treatment of Monomac-1 cells with 180 μg/mL and 270 μg/mL. Significance relative to the control was reported with * indicating a *p*-value: 0.01 < *p* < 0.05, ** indicating a *p*-value: 0.001 < *p* < 0.01, and *** indicating a *p*-value: 0.0001 < *p* < 0.001. Detailed information of western blot can be found at Figure S1.

Western blot analysis was then performed to determine the apoptotic signaling pathway induced by MMLE on Monomac-1 cell line treated with 2 different concentrations, before and after the IC50, for 24 h. Upon treatment of Monomac-1 cells with 270 μg/mL of MMLE, an upregulation of cleaved PARP (c-PARP) was observed (Figure 5). This treatment also induced a downregulation of the anti-apoptotic protein Bcl-2, coupled to an upregulation of the pro-apoptotic protein Bax indicating an increase in the Bax/Bcl-2 ratio (Figure 5) leading to apoptosis. This was further confirmed by the observed dose-dependent upregulation of cytochrome-c upon treatment of Monomac-1 with MMLE (Figure 5).

### 3.5. M. pseudolavatera Leaf Extract Induces Oxidative Stress in AML Cell Lines

**Figure 6:**
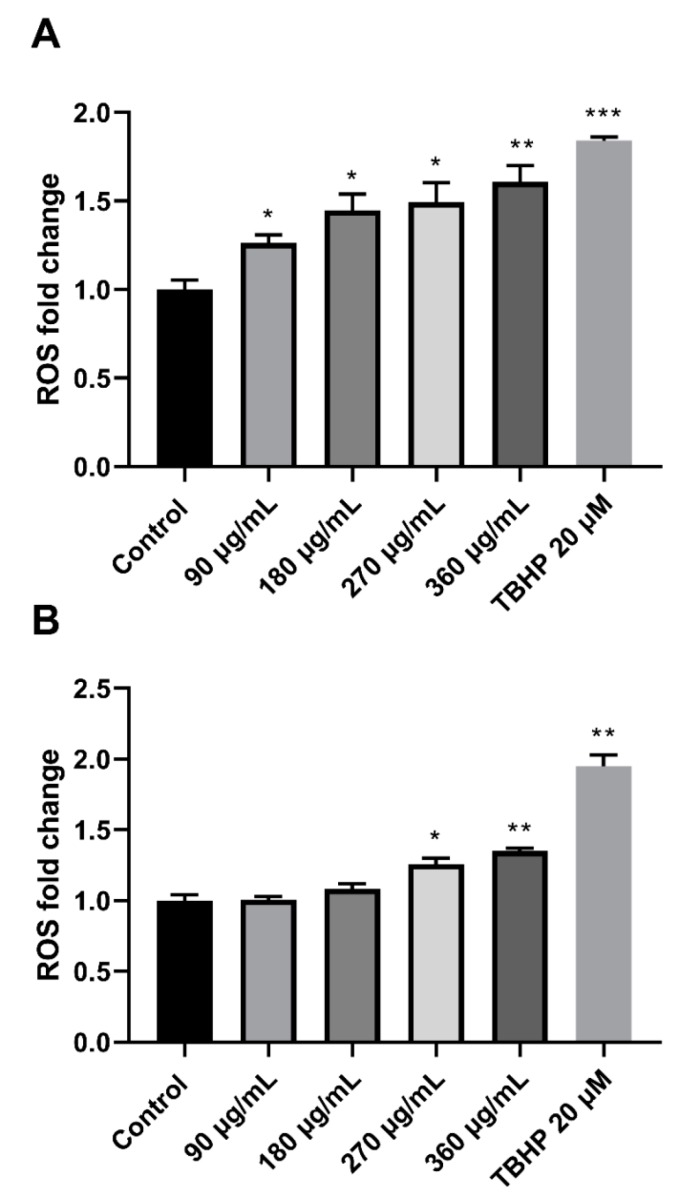
Fold change of ROS in Monomac-1 (**A**) and KG-1 cells (**B**) treated with increasing concentrations of MMLE and a positive control treated with 20 μM of TBHP. ROS levels increased significantly with increasing concentration of MMLE for both Monomac-1 (A) and KG-1 cells (B). Significance relative to the negative control was reported with * indicating a *p*-value: 0.01 < *p* < 0.05, ** indicating a *p*-value: 0.001 < *p* < 0.01, and *** indicating a *p*-value: 0.0001 < *p* < 0.001.

Reactive oxygen species levels (ROS) in Monomac-1 and KG-1 treated with increasing concentrations of MMLE were quantified using the DCFDA Cellular ROS Detection Assay kit. The recorded ROS levels showed a significant upregulation reaching 1.608-fold increase and 1.351-fold increase at 360 μg/mL for Monomac-1 and KG-1, respectively (Figure 6). This indicates that the extract is inducing oxidative stress by production of excess ROS in AML cell lines.

### 3.6. Chemical Elucidation of M. pseudolavatera Leaf Extract Using GC-MS

**Figure 7:**
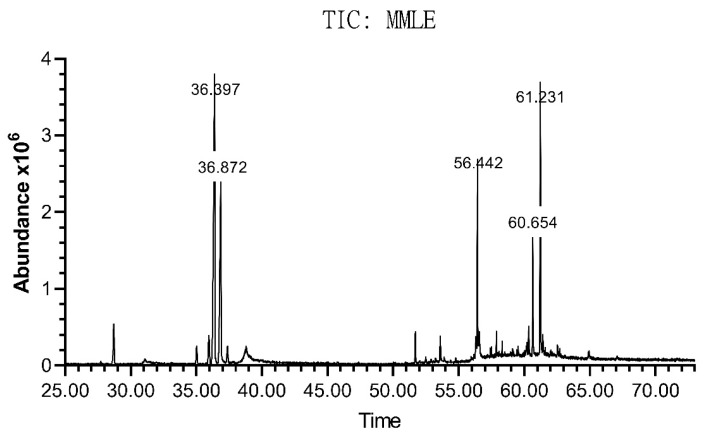
Chromatogram of *M. pseudolavatera* Webb & Berthel. methanolic leaf extract elucidated by GC-MS. Analysis of the different peaks obtained at different retention times with varying areas under the peak showed the presence of many compounds in different amounts.

The chemical composition of the extract was assessed using gas chromatography coupled to mass spectrometry (GC-MS) (Figure 7). Table 1 shows the major and minor constituents of the extract, some of which have been identified. The major compound (Peak 5: 31.7912%) was (Z,Z,Z)-9,12,15-octadecatrienoic acid methyl ester and another omega-3 fatty acid ester that os hexadecatrienoic acid was identified Peaks 7,10,13: (0.0463%). The second most abundant compound was phytol (Peak 6: 19.3447%). γ-sitosterol also constituted an important portion of the extract (Peak 25: 13.2396%) along with stigmasterol (Peak 24: 5.3751%).

### 3.7. Chemical Elucidation of M. Pseudolavatera Leaf Extract Using LC-MS

The chemical composition of the extract was assessed using liquid chromatography coupled with mass spectrometry (LC-MS). Table 2 shows the major identified constituents of the extract. The two major compounds detected were the amino acids DL-phenylalanine and DL-tryptophan (RT = 2.609 min and 5.583 min, respectively). Other important abundant compounds were tiliroside (RT = 23.857 min), 13-hydroperoxyoctadecadienoic acid (RT = 35.512 min), and quercitrin (RT = 18.598 min). Many other compounds were identified as well.

## 4. Discussion

Natural products from plants have been widely considered an important source for identifying drugs with anti-cancer properties since they are rich in bioactive components having multiple targets with minimal side-effects [18,27]. In fact, of all pharmaceutical drugs present on the market, one-third are plant-derived [28], and many chemotherapeutic drugs which have been isolated from plants are now used as standard-of-care in cancer treatment regimens. In this study, the methanolic leaf extract of *M. pseudolavatera* Webb & Berthel. was examined for its anti-proliferative and pro-apoptotic effects on AML cell lines since leaves from other Malva species have shown cytotoxic effects on other cancer types [12,13,14]. In a previous study by Solowey et al. (2014), the ethanolic extract from *Urtica membranacea* showed potent anti-cancer effects at 750 μg/mL and 1500 μg/mL and these concentrations showed to be therapeutically correlated to a mouse breast cancer model with no side-effects [29]. These concentrations are higher than the concentrations of MMLE used to treat AML cells for 24 and 48 h. For the rest of this study, all experiments were performed with 24 h incubation of the extract in order to elucidate the mechanism of action of the extract in inhibiting cancer cell proliferation, although therapeutic levels are best reached after 48 h of incubation.

Moreover, the inhibitory effect of MMLE on the proliferation of the AML cell lines was significantly stronger than its effect on the growth of the MSCs and MNCs which exhibited resistance to the extract with no significant toxicity. These results supported the promising effects of the extract in selectively targeting cancerous cells with minimal if any effects on normal healthy cells, which is a major advantage of an effective chemotherapeutic drug exhibiting target selectivity [30].

An important mechanism by which chemotherapeutic drugs achieve their selective cytotoxicity is by activating apoptosis or programmed cell death [31]. Many hallmarks of apoptosis were detected in AML cell lines treated with MMLE among which were both membrane and nuclear changes typically detected by flow cytometry and protein blots [32].

Another characteristic of apoptosis alongside DNA fragmentation and membrane moieties flipping is the cleavage of poly(ADP-ribose) polymerase (PARP) [33]. PARP was previously reported to play an important role in salvaging cells suffering from DNA damage because it is involved in DNA repair. In fact, the cleaved fragment of PARP-1 binds to double strand breaks in the DNA preventing DNA repair machinery from accessing the damage leading to apoptosis. So, upregulation of cleaved PARP (c-PARP) in Monomac-1 treated with MMLE implies that DNA repair is no longer occurring, which promotes cell death via apoptosis induction [34].

Bcl-2 and Bax are also involved in the control of cell survival by decreasing and increasing the permeability of the outer mitochondrial membrane, respectively [35,36]. The reported increase in the Bax/Bcl-2 ratio upon MMLE treatment confirms the increase in mitochondrial membrane permeability. This promotes the release of cytochrome-c from the mitochondria, explaining its upregulation, which is essential for the activation of caspases leading to apoptosis [37]. In fact, exploiting chemotherapeutic effects on mitochondrial membrane leakage was shown to be effective in selectively triggering apoptosis in cancer cells since metabolic reprogramming is an inherent step required for hyperproliferation of cancer cells [38].

Another aspect of apoptotic cell death involves reactive oxygen species (ROS). Excess cellular ROS levels cause oxidative stress which damages proteins, DNA, and cellular membranes and activates death-receptor-mediated or mitochondrial apoptotic pathways [39]. However, a previous study showed that aqueous extract from *M. pseudolavatera* Webb & Berthel. possesses antioxidant properties by scavenging free radicals [9]. The differential effect of MMLE can be attributed to variation in its concentration, implying its dose-dependent activity. In fact, in similar medicinal plants with anti-cancer properties, extracts were shown to exhibit antioxidant activity at low concentration of the extract, without achieving any cytotoxicity. At high concentrations, that same extract was shown to be cytotoxic and induced ROS [40]. In MMLE, an antioxidant compound(s) may be present in high concentrations and hence act to destroy the mitochondrial membrane and generate ROS. This mechanism was described in green tea extract with the phenolic compound epigallocatechin gallate that can decrease lipid peroxidation and enhance antioxidant capacity in hepatocytes at low concentrations and destroy the mitochondrial membrane and generate intracellular oxidative stress at high concentrations [41].

The examination of the extract composition by GC-MS and LC-MS provided insight into some of the potential compounds in MMLE responsible for its pro-apoptotic effects. Abundant identified molecules included amino acids like-phenylalanine (Phe) and tryptophan (Trp) (detected by LC-MS) as well as some of their acetylated derivatives. These two essential amino acids were not previously shown to possess such activity. Omega-3 fatty acid esters (detected by GC-MS) and these two amino acids (phe and trp) were previously shown to slow the growth of many types of cancers and increase patient outcomes when included in a standard regimen of chemotherapy since they increase the sensitivity of the cells to the conventional therapies. They also exhibit selective toxicity on cancer cells of various types but not on normal cells [42,43]. This could explain the selective toxicity seen on AML cell lines and not on MSCs and MNCs. Also, (13S)-hydroperoxyoctadecadienoic (13-HPODE) detected by LC-MS, is a linoleic acid derivative previously shown to inhibit growth of a chronic myeloid leukemia cell line K-562 [44]. In fact, the mechanism of action described was ROS-mediated and caspase-dependent apoptosis which could explain the effects observed by MMLE on AML cell lines.

Phytol, another major compound detected by GC-MS, is a diterpene alcohol and it was previously found to inhibit the growth of many cancer cell types, among which acute T-cell lymphoblastic leukemia (Molt-4) cells in a dose- and time-dependent manner. The effects of phytol were attributed to apoptosis demonstrated by DNA fragmentation and formation of apoptotic bodies. In other studies, phytol was found to have a synergistic effect with some used chemotherapeutic drugs like β-caryophyllene, in addition to inducing apoptosis in epidermoid carcinoma cells (A431) and immortalized keratinocytes (HaCaT cells) by ROS induction, activation of the apoptotic pathway involving the release of cytochrome-c, the activation of the caspase pathway, cleavage of PARP, and an increase in the Bax/Bcl-2 ratio [45,46]. These previously reported effects are consistent with the current observed morphological and molecular changes in AML cell lines upon treatment with MMLE. Another detected alcohol is quercitrin (by LC-MS). It is a plant-derived polyphenol found to enhance the effect of topotecan in breast cancer cell lines [47,48]. It was also found to reduce the cytotoxicity and genotoxicity of topotecan in bone marrow cells of mouse models in a dose-dependent manner [49].

Another important phytochemical detected by LC-MS is tiliroside. It is a glycosidic flavonoid present in many edible plants [50]. It was found to be cytotoxic against human CML cell line K-562 [51] and to inhibit cell proliferation and induce apoptosis via the extrinsic pathway in breast cancer cell lines MCF-7 and T47D [52].

Phytosterols, particularly γ-sitosterol and stigmasterol, are also important constituents of the extract (detected by GC-MS). γ-sitosterol was previously shown to be cytotoxic against colon and liver cancer cell lines by downregulating c-myc and inducing apoptosis [53]. Moreover, stigmasterol was formerly studied for activating apoptosis in hepatocellular carcinoma cell lines through upregulation of the Bax protein and downregulation of the Bcl-2 protein [54]. In another study, stigmasterol inhibited the proliferation of gastric cancer cell lines through a mitochondrial pathway [55], in accordance with the effects MMLE exhibited on AML cell lines in this study.

All these findings suggest that MMLE contains many compounds which can potentially be acting together as cancer growth inhibitors through inducing ROS and activating apoptosis in AML cell lines. Many compounds detected by GC-MS and LC-MS were not previously known for their effects on cancer cell lines and many other compounds were not identified. This should also be taken into account when explaining the pro-apoptotic effect of MMLE on AML cells.

## 5. Conclusions

In conclusion, *Malva pseudolavatera* Webb & Berthel. methanolic leaf extract showed a promising selective anti-proliferative and pro-apoptotic effect on acute myeloid leukemia cell lines, by cleaving PARP, releasing cytochrome-c, and increasing the Bax/Bcl-2 ratio. Chemical analysis of the extract showed that it is a ROS inducer and that it contains many compounds that are potentially anti-cancer compounds. Future work aims at exploring the effect of the extract on other types of cancer cells, fractionating the extract to identify the compounds with highest biological therapeutic activity and confirming the efficacy of the extract in vivo.

## Figures and Tables

**Table 1:** Table showing the composition of the *M. pseudolavatera* Webb & Berthel. methanolic leaf extract as elucidated by GC-MS.

Peak	RT	Compound	%MMLE
**1**	5.751	Unidentified A	0.2141
**2**	28.7101	Hexadecanoic acid methyl ester	3.4385
**3**	35.0314	Unidentified B	1.4208
**4**	35.9573	(E,E)-9,11-Octadecadienoic acid methyl ester	2.7188
**5**	36.3974	Methyl linolenate	31.7912
**6**	36.8717	Phytol	19.3447
**7**	37.3804	Octadecanoic acid methyl ester	1.3903
**8**	38.7407	Unidentified C	0.124
**9**	38.7864	7,10,13-Hexadecatrienoic acid methyl ester	0.0463
**10**	38.8093	Unidentified C	0.0408
**11**	51.7034	2,2′-methylenebis [6-(1,1-dimethylethyl)-4-methyl-phenol	1.6741
**12**	53.607	Unidentified D	1.47
**13**	56.3158	Unidentified E	1.4302
**14**	56.4415	(Z,Z,Z)-9-(3-hexenylidenecyclopropylidene)-,2-hydroxy-1-(hydroxymethyl)nonanoic acid, ethyl ester	8.0675
**15**	56.584	Unidentified F	1.5
**16**	57.4074	Unidentified G	0.2354
**17**	57.505	Unidentified H	0.3
**18**	57.8875	Cyclotetracosane	0.6203
**19**	58.322	Unidentified I	0.31
**20**	59.5393	α-Tocopherol	0.3324
**21**	60.168	Unidentified J	0.3748
**22**	60.2195	Unidentified K	0.9207
**23**	60.3509	3-β-Ergost-5-en-3-ol	1.5331
**24**	60.6538	Stigmasterol	5.3751
**25**	61.2311	γ-Sitosterol	13.2396
**26**	61.4254	3-methoxy-19-Norpregna-1,3,5(10)-trien-17-ol	1.2401
**27**	64.94	Unidentified L	0.39

**Table 2:** Table showing the composition of the *M. pseudolavatera* Webb & Berthel. methanolic leaf extract as elucidated by liquid chromatography coupled with mass spectrometry (LC-MS).

RT	Compound	Area Max
2.609	DL-phenylalanine	1.21 × 10^6^
5.583	DL-tryptophan	1.10 × 10^6^
23.857	Tiliroside	4.25 × 10^5^
10.893	5-[(6,7,8-trimethoxy-4-quinazolinyl)amino]pentyl nitrate	3.12 × 10^5^
35.512	13-hydroperoxyoctadecadienoic acid	1.95 × 10^5^
14.915	*N*-acetyl-*L*-phenylalanine	1.12 × 10^5^
18.865	3-amino-2-pyrazinecarboxylate	1.04 × 10^5^
18.598	quercitrin	7.35 × 10^4^
13.603	*N*-(4-{methyl[(1-methyl-1*H*-pyrazol-4-yl)methyl]sulfamoyl}phenyl)acetamide	7.21 × 10^4^
26.441	Decyl hydrogen sulfate	6.97 × 10^4^
14.775	Suberic acid	6.73 × 10^4^
19.007	9-hydroxynonanoic acid	6.10 × 10^4^
16.791	*L*-acetyltryptophan	5.12 × 10^4^
14.082	5-(benzyloxy)-2-piperazinopyrimidine	5.05 × 10^4^

## Supplementary Materials

The following are available online at https://www.mdpi.com/2072-6694/12/2/435/s1, Figure S1: Western blot analysis and quantification of expression levels of apoptosis-regulating proteins in Monomac-1 cells treated with MMLE for 24 h.
