# Supplementary material for: Malva pseudolavatera Leaf Extract Promotes ROS Induction Leading to Apoptosis in Acute Myeloid Leukemia Cells In Vitro
Source: Cancers (Basel). 2020 Feb 13;12(2):435. doi: 10.3390/cancers12020435 (PMC7072199; doi:10.3390/cancers12020435)
Supplement: Supplementary file 1 [file cancers-12-00435-s001.pdf]

Supplementary Materials

Malva pseudolavatera Leaf Extract Promotes ROS Induction Leading to Apoptosis in Acute Myeloid Leukemia Cells In Vitro

Marianne El Khoury, Tony Haykal, Mohammad H. Hodroj, Sonia Abou Najem, Rita Sarkis, Robin I. Taleb and Sandra Rizk

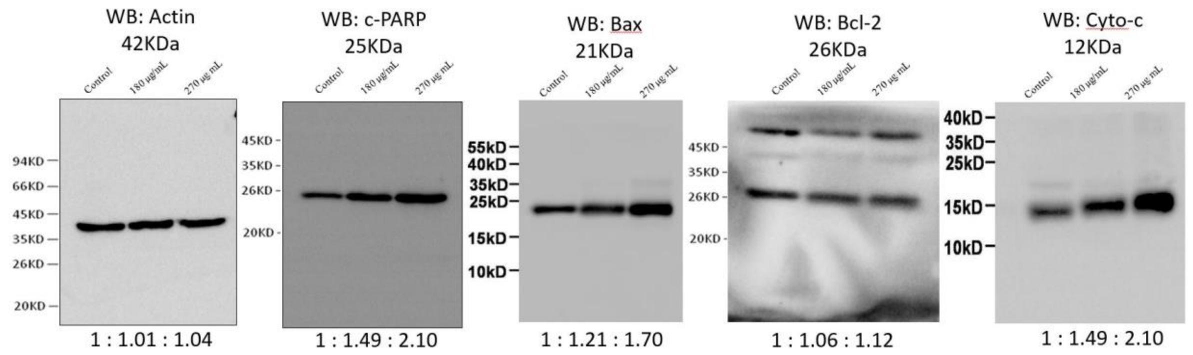

Figure S1. Western blot analysis and quantification of expression levels of apoptosis-regulating proteins in Monomac-1 cells treated with MMLE for 24 h.

© 2020 by the authors. Licensee MDPI, Basel, Switzerland. This article is an open access article distributed under the terms and conditions of the Creative Commons Attribution (CC BY) license (http://creativecommons.org/licenses/by/4.0/).
